# Aberrant DNA Methylation Mediates the Transgenerational Risk of Metabolic and Chronic Disease Due to Maternal Obesity and Overnutrition

**DOI:** 10.3390/genes12111653

**Published:** 2021-10-20

**Authors:** Yan Li, Carol A. Pollock, Sonia Saad

**Affiliations:** 1 Hospital of Chengdu University of Traditional Chinese Medicine, Chengdu 610072, China; yanli@stu.cdutcm.edu.cn; 2 Kolling Institute of Medical Research, University of Sydney, Sydney, NSW 2065, Australia; carol.pollock@sydney.edu.au

**Keywords:** DNA methylation, maternal obesity, overnutrition, metabolic diseases, offspring

## Abstract

Maternal obesity is a rapidly evolving universal epidemic leading to acute and long-term medical and obstetric health issues, including increased maternal risks of gestational diabetes, hypertension and pre-eclampsia, and the future risks for offspring’s predisposition to metabolic diseases. Epigenetic modification, in particular DNA methylation, represents a mechanism whereby environmental effects impact on the phenotypic expression of human disease. Maternal obesity or overnutrition contributes to the alterations in DNA methylation during early life which, through fetal programming, can predispose the offspring to many metabolic and chronic diseases, such as non-alcoholic fatty liver disease, obesity, diabetes, and chronic kidney disease. This review aims to summarize findings from human and animal studies, which support the role of maternal obesity in fetal programing and the potential benefit of altering DNA methylation to limit maternal obesity related disease in the offspring.

## 1. Introduction

The global epidemic of obesity has become a widespread serious health issue among children, adolescents, and adults [1]. The World Health Organization has reported that ~20 percent of adults will be obese by 2025 worldwide and obesity currently causes ~38.6 million people’s death and disability each year due to musculoskeletal disorders; cardiovascular diseases; and mental disorders such as depression, anxiety, and stress [2,3]. In addition, statistical reports showed the prevalence rate of obesity in women of bearing age in the United States is 31.8%, with approximately 60% of women reported as being overweight during the gestation period [4,5]. It is known that oocyte quality declines in obese women, and both obese mothers and their offspring are liable to health problems [6]. Specifically, during pregnancy and lactation, obese mothers are susceptible to maternal–fetal metabolic complications compared to mothers with normal weight [7]. In addition, evidence indicates that offspring born to obese mothers are predisposed to a higher birth weight, higher body mass index (BMI), and more fat accumulation, which are recognized to contribute to metabolic diseases in future life [8]. Maternal obesity correlates with chronic inflammation, increased insulin resistance, and hyperlipidemia [9]. Consequently, excess lipid exposure may have detrimental effects on embryonic and fetal development. Increased fat serum levels in the mothers during gestation can induce inflammation and alter lipid and glucose metabolism, eventually leading to an adverse effect on the offspring [10].

Various studies have showed that maternal obesity can either affect fetal development in utero or have persistent adverse effects on the phenotypes of multiple generations via epigenetic changes, heritable and environmental processes, which alter downstream gene transcription but without altering DNA sequence [11]. Indeed, obese mothers may transmit their metabolic phenotypes to their offspring which enhances their risk for chronic metabolic diseases [12] and at least, in part, explains the rapid increase in prevalence rate of metabolic and chronic diseases. In addition, epigenetic modification, which is known to be regulated by the intrauterine environment [13], can persist over consecutive generations, which results in long-term impacts to the offspring [14]. The majority of these epigenetic changes appear in utero during the important periods of fetal growth and may play a subsequent role in fetal programing [15]. Epigenetic modification includes DNA methylation and acetylation, RNA and histone modification, as well as regulation by non-coding RNAs (ncRNAs) [16]. Among those, DNA methylation is one of the most widely observed epigenetic change in maternal and fetal programming [17] and thus its role in maternal obesity related disease on the offspring will be covered in this review.

## 2. Location, Writers, Erasers, Readers of DNA Methylation

**Figure 1:**
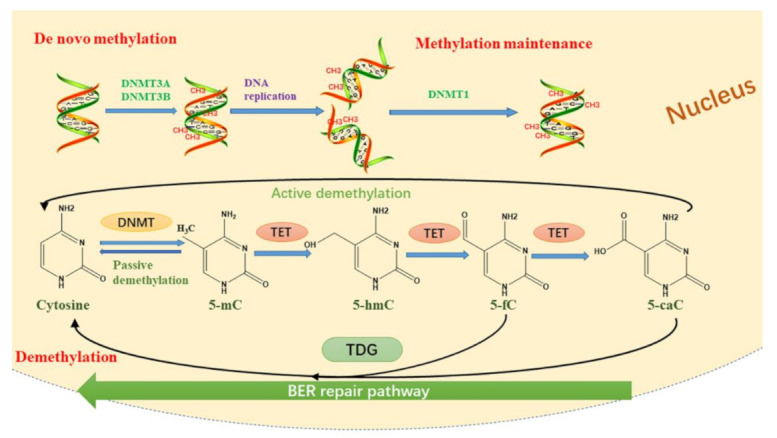
The dynamic and reversible processes of DNA methylation. The whole epigenetic progression of 5mC contains three aspects: de novo methylation, methylation maintenance, and demethylation. *DNMT3A* and *DNMT3B* were reported to be involved in de novo methylation, while *DNMT1* was found to participate in the maintenance of DNA methylation. Besides, the process of demethylation can be divided into two parts: passive demethylation and active demethylation. Passive DNA demethylation is attributed to the absence of DNMTs, while active demethylation is due to the involvement of *TET* or *TDG* enzymes and BER repair pathway. Abbreviation: DNA methyltransferase: *DNMT*; Ten eleven translocation: *TET*; Thymine DNA glycosylase: *TDG*; Base excision repair: BER; Fifth position of cytosine: 5mC; 5-hydroxymethylcytosine: 5hmC; 5-formylcytosine: 5fC; 5-carboxylcytosine: 5caC.

DNA methylation is a universal and heritable epigenetic modification that mainly induces transcriptional repression [18]. DNA modification can theoretically occur in any of the DNA bases, although only modifications of cytosine and adenine are described to date [18]. Most of the DNA methylation occurs on cytosines nucleotide that precede a guanine nucleotide or CpG sites. Methylation of the fifth position of cytosine (5 mC) is the most studied DNA methylation [19]. Overall, CpG sites in mammalian genomes are relatively rare, which may be because 5mC can easily deaminate to thymine [20]. With the exception of CpG islands (regions of the genome that contain a large number of CpG repeats) that are mainly situated in the promoter and exon regions [21,22], the remaining CpG sites across the genome are highly methylated [21]. Methylated CpG islands recruit methyl-binding proteins, which result in stable silencing of gene expression via inhibition of transcription factor binding [23]. Although hypermethylation largely decreases the gene transcriptome level, one study has reported that DNA methylation in intragenic regions (including gene body, 5′and 3′ untranslated regions) may increase the transcriptome by blocking the initiation of alternative promoters [24]. Enzymes which participate in DNA modification are categorized as ‘writers’ (methylation), ‘erasers’ (de-methylation), and ‘readers’ (recognition). ‘Writers’ are the enzymes that play an important role in establishing and maintaining methylation. ‘Erasers’ can catalyze the removal of the methyl donors. ‘Readers’ can interpret DNA methylation and finally affect transcription [25] (Figure 1).

### 2.1. Writers

DNA methyltransferases (*DNMTs*), including *DNMT1*, *DNMT3A*, *DNMT3B*, and *DNMT3L*, directly promote S-adenosyl methionine contribution to DNA methylation, specifically at CpG sites [26,27]. Although these enzymes share a similar structure, they have different functions and expression profiles [28]. *DNMT1* methylates hemi-methylated CpGs and maintains the methylation in daughter cells [29]. Additionally, *DNMT1* also has the ability to repair DNA methylation [28]. *DNMT 3a* and *DNMT 3b* function as de novo *DNMTs*, which methylate both native and synthetic DNA without preference for hemi-methylated DNA [30]. They are responsible for setting up genomic imprints and methylation patterns during germ cell development and embryogenesis [31].

### 2.2. Erasers

DNA demethylation can be divided into passive and active demethylation. Passive DNA demethylation occurs when *DNMTs* are reduced or absent [32]. Active DNA demethylation occurs by deamination or oxidation reactions and involves specific enzymes [33]. The ten-eleven translocation (*TET*) enzymes can catalyze the demethylation of 5mC and cause the reversal of methylation [34]. Activation-induced deaminase (*AID*)/*APOBEC* enzymes can cause 5mC or 5-hydroxymethylcytosine (5hmC) to deaminate to uracil. Several intermediate products can be replaced by cytosine via the base excision repair (*BER*) pathway initiated by uracil DNA glycosylase (*UDG*) family, such as thymine DNA glycosylase (*TDG*), single-strand-selective monofunctional uracil DNA glycosylase (*SMUG1*), and methyl-CpG-binding domain (*MBD*)4 [35] (Figure 1). Ultimately, 5mC oxidized bases, such as 5-hydroxymethyluracil (5hmU), 5-formylcytosine (5fC), as well as 5-carboxylcytosine (5caC), are recognized and excised then finally replaced by a bare cytosine regulated by *TDG* and *SMUG1* [33]. Active DNA demethylation involves multiple enzymes and a complex process which requires further investigation.

### 2.3. Readers

DNA methylation is recognized by three separate kinds of proteins: the 5- methylcytosine binding domain (*MBD*) family; the ubiquitin-like, containing plant homeodomain and ring finger domain (*UHRF*) family; and the zinc-finger family. Among them, *MBD* family has a conservative region for the readout of DNA methylation and recruitment of methylases to methylated DNA [36]. The *UHRF* proteins bind to *DNMT1* and then target it to hemi-methylated DNA to maintain DNA methylation [36]. Zinc-finger proteins including Kaiso, *ZBTB4*, and *ZBTB38*, like the *MBD* family, binds methylated DNA and repress transcription [37].

## 3. DNA Methylation and Maternal Obesity-Related Offspring Predisposition to Future Disease

The genome in early mouse embryo is significantly methylated. However, during the stage of preimplantation, a wave of genome-wide demethylation occurs followed by de novo methylation in somatic and extra-embryonic tissues [38]. In the progression of differentiation, methylation patterns are altered in a lineage-specific fashion. During fetal development, a second wave of demethylation occurs and is accompanied by re-establishment, especially in primordial germ cells [39,40]. DNA modification patterns are affected by the maternal environment and are formed during the periods of embryogenesis, fetal growth or early postnatal life [38]. Therefore, alteration of gene methylation in utero or during the critical period of development, which ranges from pre-conception to early childhood, can lead to permanent structural changes in an organ and development of disease in the offspring.

Maternal obesity has previously been shown to induce chronic disease in the offspring. First, maternal obesity can affect the weight of the offspring leading to obesity and increased susceptibility to chronic diseases later in life [41]. Second, maternal obesity may predispose the offspring to chronic diseases through intrauterine mechanisms, for example reshaping the uterine immune cell landscape [42]. Moreover, chronic diseases induced by maternal obesity can be transmitted to offspring through epigenetic regulation. Indeed, recent studies have indicated that maternal obesity results in the alteration of DNA methylation, histone modification as well as noncoding RNAs in oocytes and sperm [43]. A further study showed that 56 CpGs sites were significantly and differentially methylated in placental tissue of overweight mothers compared to normal weight mothers [44].

Epidemiological studies have shown that maternal obesity or overnutrition during pregnancy or lactation exerts a lasting effect on the prevalence of metabolic diseases in the next generation, including non-alcoholic fatty liver disease (NAFLD), obesity, diabetes, as well as chronic kidney disease (CKD) [45,46,47]. Human and animal studies also showed that maternal obesity or overnutrition during conception or lactation can modulate DNA methylation of different genes involved in energy metabolism, glucose homeostasis, insulin signaling and fat deposition, which support the role of DNA methylation in maternal obesity-induced risk of NAFLD, obesity, diabetes, and CKD (Table 1) [48,49,50,51,52,53].

### 3.1. DNA Methylation and Maternal Obesity-Related Offspring Predisposition to NAFLD

Previous studies have elucidated that intrauterine exposure to maternal obesity disrupts liver metabolic programming, characterized by alterations in hepatic fatty acid oxidation and in the tricarboxylic acid cycle and lipid metabolism [84,85]. Maternal obesity increases insulin and leptin resistance, dyslipidemia, hepatic inflammation, and steatosis in subsequent generation [85,86,87,88,89]. The effect of maternal obesity on the offspring can last over multiple generation. Indeed, offspring born to obese mothers tend to develop obesity and hepatic steatosis over three subsequent generations [12,14].

Epigenetic studies showed that exposure to obesity in utero, also resulted in hepatic hypermethylation and this was associated with alteration in DNMTs expression and enzymatic activity [90]. Epigenetic changes exert a lasting effect, particularly via DNA methylation of genes associated with mitochondrial function and lipid metabolism [91]. Maternal obesity was found to alter the methylation level of hepatocyte nuclear factor-4 alpha (*HNF4A*), Peroxisome proliferator-activated receptor gamma coactivator (*PPARGC*)-*1β* as well as fibroblast growth factor (*FGF*)-*21* in mice offspring liver, which have crucial roles in hepatic fibrosis and lipid accumulation [92,93]. Patients with NAFLD have global DNA methylation in hepatic tissues in association with differential levels of PPARGC-1A compared to healthy liver samples [94]. In addition, maternal obesity and maternal overnutrition are associated with Leptin hypermethylation and peroxisome proliferator-activated receptor (PPAR)α hypomethylation in the tissue of offspring’s oocytes and liver [54,55]. *PPAR α* and liver X receptor *α* (*LXRα*) which are involved in the metabolism of several important lipids, are significantly hypermethylated in the liver tissues of mice offspring born to obese mothers [56,57]. Moreover, *Lipin 1*, a gene involved in lipid generation, was hypermethylated in the transcription factor binding sites of the offspring’s liver tissue as a result of maternal obesity [58]. The biological processes included regulation of sterol regulatory-element binding protein (*SREBP*) signaling, phospholipid transport, as well as granulocyte differentiation, which are implicated in controlling lipogenesis and lipid uptake, and are known to contribute to the development of NAFLD and nonalcoholic steatohepatitis [95]. DNA methylation levels in the promoters of the glycerol-3-phosphate acyltransferase 1 (*GPAT1*) is lower and the transcriptome level of *GPAT1* and *SREBP-1* are higher in the offspring of obese mothers compared to offspring of normal weight mothers, in association with increased hepatic triglyceride levels [59,60] also suggesting increased risk of hepatic steatosis in the offspring [96]. Peng et al. recently demonstrated that offspring born to mothers who had a high-fat diet displayed a disruption of lipid homeostasis, which is accompanied by altered methionine and abnormal one-carbon metabolism in offspring livers. This would lead to DNA hypermethylation and L-carnitine depletion associated with deactivation of AMP-activated protein kinase (*AMPK*) signaling and decreased expression of PPAR-α and genes for fatty acid oxidation [97].

Offspring exposure to maternal obesity and maternal overnutrition also induced glucose-regulated protein (*GRP*)-*78* hypermethylation in association with downregulation of gene expression [61]. Reduced levels of *GRP78* gene expression increases the activity of unfolded protein response (*UPR*) [98], which has been reported in hepatic dyslipidemia and NAFLD [99,100,101]. Furthermore, platelet-derived growth factor receptor (*PDGFR*)-*β*, proinflammatory and profibrogenic regulator, which can act as a potential target in diagnosing and treating early stages of NAFLD fibrosis [62], was hypomethylated and upregulated transcriptionally in the offspring of obese mothers [62]. Collectively, these data strongly support the role of DNA methylation in the development of NAFLD in the offspring exposed to maternal obesity in utero.

### 3.2. DNA Methylation and Maternal Obesity Related Offspring Predisposition to Obesity and Metabolic Disease

Maternal obesity and overnutrition during prenatal and postnatal periods have a negative influence on offspring lipid metabolism and predisposes the offspring to increased fat mass, chronic inflammation, and oxidative stress. This effect is further amplified if the offspring consume high fat and calorie diet after the lactation period [102,103,104,105]. Therefore, the increased prevalence of maternal obesity and maternal overnutrition correlates with the risk of obesity in offspring [45,92,106].

Various studies have discovered that obesity, known as a common metabolic disease, is associated with epigenetic alterations especially DNA methylation [107,108]. Several imprinted genes involved in metabolic diseases such as *PPARα* [54], *IGF2* [109], *H19* [109], and paternally expressed gene 3 (*Peg3*) [110] are differentially methylated in the mammalian gametes in oocytes of control and obese animals and their offspring, which suggests that maternal obesity can induce transgenerational inheritance of metabolic disease through DNA methylation. Exposure to maternal overnutrition or maternal obesity before or during gestation or lactation, leads to an incremental increase in the mRNA level of several adipogenic genes in peripheral fat in fetal sheep such as *PPARG*, fatty acid synthase, lipoprotein lipase, adiponectin, and leptin which participate in energy and lipid metabolism [63,64,67]. Alteration in DNA methylation levels of those genes were also demonstrated in the visceral fat of mice offspring due to maternal obesity [111]. Furthermore, studies on rodents have revealed that maternal obesity leads to increased birth weight, increased leptin levels, and hypermethylation of pro-opiomelanocortin (*POMC*) in the promoter regions of the offspring, which has a vital role in leptin resistance [65,66]. Previous studies also observed that maternal obesity may influence the prevalence of offspring obesity, which may be mediated by the methylation of promoter DNA of three key genes related to metabolic syndrome (*PPARGC1A*, *PPARG*, and mitochondrial transcription factor A (*TFAM*)), as identified in umbilical cord blood [52,67].

Additionally, offspring born to obese mothers have decreased gene methylation of key adipogenic transcription regulators, including CCAAT/enhancer binding protein beta (*C/EBP-β*) and zinc-finger proteins, which may result in elevated adipogenic tissue differentiation during embryonic and fetal growth periods and predispose to metabolic disorders [68,69]. DNA methylation array demonstrated that genes related to fatty acid oxidation (*PRKAG2*, *ACC2*, *CPT1A*, *SDHC*) were hypermethylated in the cord blood mesenchymal stem cells (MSCs) of obese mothers, which was positively associated with infant adiposity [70]. Moreover, MSCs in umbilical cord blood of obese mothers displayed increased lipid deposition, decreased fatty acid oxidation, in association with disrupted *AMPK* signaling [70]. Additional studies have found that the TAP-binding protein (*TAPBP*) is hypermethylated in umbilical cord blood of obese mothers, which suggests that maternal obesity can result in the development of obesity in the offspring via reducing tapasin (decreased tapasin can lower CD8 + T-cell responses in vitro) leading to impaired immune responses in offspring [71,72]. In addition, aryl hydrocarbon receptor repressor (*AhRR*) was hypermethylated in the umbilical cord of obese mothers compared to lean mothers. *AhRR* functions as an inhibitor of adipocyte differentiation by negatively regulating *PPARG* during adipogenesis. Collectively, these data suggest that offspring of obese mothers are at increased risk of obesity and metabolic disease [73,74,75]. Therefore, targeting DNA methylation during the early postnatal period might provide a novel preventative measure for limiting transgenerational obesity.

### 3.3. DNA Methylation and Maternal Obesity Related Offspring Predisposition to Diabetes Mellitus

Epidemiological studies demonstrate that exposure to obesity during the prenatal and postnatal periods increases the risk of offspring to diabetes [112,113], as insulin resistance may be programmed in utero and exposure to insulin resistance in early life increases the risk of diabetes [114].

Maternal obesity and overnutrition results in epigenetic alterations of insulin-signaling molecules [115,116]. Genes involved in type 1 diabetes mellitus, such as human leukocyte antigen (*HLA*)-*DQA1*, *HLA-DQB1* were hypermethylated in the promoter region of whole blood sample from offspring born after maternal bariatric surgery compared to before bariatric surgery, and genes involved in type 2 diabetes mellitus includes *POMC*, *IGF2*, insulin receptor (*INSR*), fat mass- and obesity-associated protein (*FTO*) as well as tumor necrosis factor (*TNF*), were either hypermethylated (*POMC*, *IGF2*, and *INSR*) or hypomethylated (*FTO* and *TNF*) [76,77]. In addition, several genes involved in the immune/inflammatory pathways were differentially methylated in the peripheral blood of offspring born to obese women before bariatric surgery compared with siblings born after weight loss surgery [117]. Genes involved in immunological processes (including *TNF -α*, interleukin, and major histocompatibility complex (*MHC*) class I and II signaling pathways) were also differentially methylated in offspring of obese mothers. These genes correlate with β-cell mass restriction as well as insulin resistance [78,79]. Several genes involved in immune responses in patients with type 1 diabetes were hypermethylated in umbilical cord blood-derived monocytes, including signal transducer and activator of transcription 1 (*STAT1*), T cell receptors (*CD247*, *CD28*, and *CD3E*), MHC I class or II subunits (*HLADMB* and *HLA-DQB1*) [79].

In addition to its role in obesity, leptin is also known to have a role in diabetes [118] and may be implicated in energy homeostasis and insulin resistance induced by epigenetic regulation [80]. Many studies reported that leptin promoters were hypermethylated in the placentas of obese mothers leading to decreased placental leptin expression while leptin deficiency is associated with hyperglycemia both in humans and animals [80,81,82]. Moreover, maternal nutrition restriction leads to the hypomethylation of phosphoenolpyruvate carboxykinase 1 (*PCK1*) (a rate-limiting hepatic gluconeogenic enzyme) in the promoter regions with a concomitant increment in transcriptome expression in fetal and postnatal liver [115,116,119,120]. *IGF2* was overexpressed in the islets of F1 and F2 generations and was hypermethylated in different CpG sites, which may cause mitochondrial dysfunction, reduction of β-cell mass and susceptibility to diabetes over multiple generations [121]. However, to date, it is not clear whether alterations in gene methylation due to maternal obesity is a causative or occur as a consequence of diabetes.

### 3.4. DNA Methylation and Maternal Obesity-Related Offspring Predisposition to Chronic Kidney Diseases

The kidney is a highly metabolic organ and is vulnerable to the effect of the intrauterine environment [122]. Although few studies have addressed the role of epigenetic regulation in maternal obesity-related CKD in the offspring, it is increasingly recognized that detrimental renal effects of maternal obesity can be transmitted to subsequent generations of obese women resulting in increased susceptibility to CKD [123,124]. Additionally, maternal obesity predisposes the offspring to dysglycemia, diabetes as well as hypertension, which, in turn can induce future renal dysfunction [125].

Epigenetic regulations were shown to contribute to the development of CKD, diabetic nephropathy, and renal fibrosis [126]. Reactive oxygen species (ROS) levels, which are strongly implicated in CKD development, are also closely linked to epigenetic changes [127]. Incremental increase in oxidative stress and mitochondrial impairment during the period of oocyte development laid down through epigenetic changes can also contribute to the transgenerational development of maternal obesity related CKD [47,83]. Sureshchandra et al. demonstrated that maternal obesity correlates with global hypomethylation of key immune genes (T cells, cytokine, chemokines) in umbilical cord blood-derived monocytes [79], some of which are involved in the development of inflammatory responses in kidney tissues and CKD pathology [128]. We have additionally demonstrated that maternal obesity induces global DNA methylation in the offspring kidney in association with established kidney fibrosis [129]. The evidence hence suggests that DNA methylation is involved in maternal obesity-induced CKD in offspring. Additional studies are required to identify the specific genes which are differentially methylated in renal tissue in the offspring of obese mothers and determine the mechanisms of fetal programming to CKD.

## 4. Epigenetic Pharmacology

DNA methylation is important for fetal development, and as discussed above, DNA hypermethylation or hypomethylation can occur in utero depending on the fetal development stage. DNA methylation, no doubt, plays an important role in the development and progression of metabolic and kidney disease, suggesting that DNA methylation modulators might have a role in disease prevention due to fetal programing. It is not clear to date whether administration of demethylating agents or supplements that induce DNA methylation during the prenatal or postnatal period or during gestation would have a detrimental or protective effect on the offspring. Whole genome methylation studies are still lacking. It is also unclear to date whether DNA methylation is causative of disease transmission due to maternal obesity or just associated with disease progression. Supplementation of methyl-donors, such as folate, choline, vitamin B12, serine, betaine, as well as methionine during gestation, regulate the production of the most important methyl-donor (S-adenosyl-methionine) as well as one-carbon metabolisms and therefore modify DNA methylation status in the offspring epigenome, leading to the alterations of gene expression [111,130,131,132,133]. In addition, having a cocktail of choline, betaine, folic acid, as well as vitamin B12 can epigenetically reduce the long-lasting effect of maternal obesity and prevent the transgenerational amplification of obesity [134,135]. Furthermore, supplementing methyl donor to lactating rats was shown to decrease maternal obesity induced hepatic adipose deposition in offspring [136], and significantly decrease blood glucose level in the next generation by downregulating the insulin resistance pathway [137]. On the other hand, we have demonstrated that administration of hydralazine, an anti-hypertensive drug, during gestation to obese dams at low dose reduces renal global DNA methylation in offspring and improves maternal obesity-induced renal fibrosis independent of blood pressure regulation [129]. Note that such effect on global DNA methylation is due to overall effects on individual genes, some of which would be differentially hyper-or hypomethylated. Regardless, altering DNA methylation during gestation and/or early postnatal period is still a challenge due to safety issues with available epigenetic modulators. In summary, the available evidence strongly supports the key role of DNA methylation in maternal obesity related disease in offspring and suggests that early intervention may be necessary to prevent/reduce the increased prevalence of metabolic and chronic disease induced by maternal obesity. Methyl supplementation or DNA demethylation agents prior to or during pregnancy may enable the change of offspring’s phenotype and prevent transgenerational amplification of maternal obesity. Such a hypothesis needs to be confirmed in animal studies before it can be extended to humans. Given that many methyl donors are extracted from different diets, maternal diet is also very crucial to prevent or reduce the development of adverse effect on the offspring due to maternal obesity.

## 5. Conclusions and Perspectives

Maternal obesity predisposes the offspring to obesity, diabetes, CKD, and NAFLD via epigenetic regulation, particularly DNA methylation.

While the data supporting the role of maternal obesity and DNA methylation in disease development are clear, we cannot exclude the additional effect of the postnatal environment such as diet, stress, physical activity, lifestyle, etc. on epigenetic modification and disease development. Current epidemiological studies that are able to attribute the independent contribution of maternal obesity to the offspring’s phenotype are limited as it is difficult to determine the impacts of prenatal exposure to high fat diet from post-natal environmental effects. It is also unclear whether maternal obesity and post-natal overnutrition have the same effect on the offspring. Few interventional studies have follow-up data focused on weight management and remedial therapies before and during the gestation period in order to reduce detrimental effects of maternal obesity on chronic disease in the next generation. Therefore, novel interventions are required to be devised and studied, together with long time longitudinal follow-up studies to assess the effect of maternal obesity on multiple generations.

Assessing DNA methylation profiles due to maternal obesity is required to shed more light on the role of DNA methylation in disease programming. Because epigenetic modifications are considered as durable but reversible processes, interventions and strategies to prevent or reverse disease development can be carried out once the mechanisms are uncovered. Additional experiments are imperative to examine whether the effect of maternal obesity on DNA methylation are transmitted through multiple generations and whether alteration of gene methylation before the onset of pregnancy or during pregnancy/early postnatal periods can safely reverse disease programing.

Considering that most women who are overweight/obese before pregnancy will remain obese throughout their gestation period, and due to the fact that obesity induces reversible epigenetic changes to the DNA, using epigenetic modulators to regulate DNA methylation before the onset of pregnancy might be beneficial in preventing adverse effect due to maternal obesity on the offspring.

In conclusion, this review demonstrated the effect of maternal obesity/overnutrition on transgenerational transmission of metabolic and kidney disease and provides evidence for the role of DNA methylation in maternal obesity-induced disease in the offspring. We demonstrated that weight management and fat diet restriction during prenatal and postnatal periods, together with methyl donor supplements or epigenetic modulators may improve the maternal metabolic status and prevent transgenerational amplification of disease predisposition.

## Figures and Tables

**Table 1:** Association of maternal obesity with offspring metabolic diseases based on the role of DNA modification in various studies.

Diseases	Major Finding	Reference
Non-alcoholic fatty liver disease (NAFLD)	Maternal obesity and maternal overnutrition are associated with Leptin hypermethylation and peroxisome proliferator-activated receptor (*PPAR*)α hypomethylation in the tissue of offspring’s oocytes and liver	[54,55]
	*PPARG* and liver X receptor α(*LXRα*) which are involved in the metabolism of several important lipids, are significantly hypermethylated in the liver tissues of mice offspring born to obese mothers	[56,57]
	Lipin 1, a gene involved in lipid generation, was hypermethylated in the transcription factor binding sites of the offspring’s liver tissue as a result of maternal obesity	[58]
	DNA methylation levels in the promoters of the glycerol-3-phosphate acyltransferase 1 (*GPAT1*) is lower and the transcriptome level of *GPAT1* and sterol regulatory element binding protein-1 (*SREBP*-*1*) are higher in the offspring of obese mothers compared to offspring of normal weight mothers, in association with increased hepatic triglyceride levels	[59,60]
	Offspring exposure to maternal obesity and maternal overnutrition also induced glucose-regulated protein (*GRP*)-*78* hypermethylation in association with downregulation of gene expression mothers	[61]
	Platelet-derived growth factor receptor (*PDGFR*)-β, a proinflammatory and profibrogenic regulator, which can act as a potential target in diagnosing and treating early stages of non-alcoholic fatty liver disease (NAFLD) fibrosis, was hypomethylated and upregulated transcriptionally in the offspring of obese mothers	[62]
Obesity	Exposure to maternal overnutrition or maternal obesity before or during gestation or lactation, leads to an incremental increase in the mRNA level of several adipogenic genes in in perirenal fat in fetal sheep. The offspring such as *PPARG*, fatty acid synthase, lipoprotein lipase, adiponectin, and leptin which participate in energy and lipid metabolism. Alteration is the DNA methylation of those genes was also demonstrated in the in visceral fat of mice offspring due to maternal obesity	[63,64]
	Studies on rodents have revealed that maternal obesity leads to increased birth weight, increased leptin levels, and hypermethylation of pro-opiomelanocortin (*POMC*) in the promoter regions of the offspring, which has a vital role in leptin resistance	[65,66]
	Previous studies also observed that maternal obesity may influence the offspring’s metabolism and increased the prevalence of offspring obesity, and this could be affected by the promoter DNA methylation of three key genes related with metabolic syndrome (*PPARGC1A*, *PPARG*, and mitochondrial transcription factor A (*TFAM*)) in umbilical cord blood	[52,67]
	Offspring born to obese mothers have decreased gene methylation of key adipogenic transcription regulators of adipogenesis, including CCAAT/enhancer binding protein beta (*C*/*EBP*-β) and zinc-finger proteins, which may result in elevated adipogenic tissue differentiation during embryonic and fetal growth periods and result in metabolic disorders	[68,69]
	DNA methylation array demonstrated that genes related to fatty acid oxidation (Protein kinase AMP-activated non-catalytic subunit gamma 2 (*PRKAG2*), acetyl-CoA carboxylase 2 (*ACC2*), carnitine palmitoiltransferase I (*CPT1A*), succinate dehydrogenase subunit C (*SDHC*)) were hypermethylated in the cord blood mesenchymal stem cells (MSCs) of obese mothers, which was positively associated with infant adiposity	[70]
	TAP-binding protein (*TAPBP*) is hypermethylated in umbilical cord blood of obese mothers, which suggests that maternal obesity can result in the development of obesity in the offspring via reducing tapasin (decreased tapasin can lower *CD8* + T-cell responses in vitro) leading to impaired immune responses in offspring	[71,72]
	Aryl hydrocarbon receptor repressor (*AhRR*) was hypermethylated in the umbilical cord of obese mothers compared to lean mothers. *AhRR* functions as an inhibitor of adipocyte differentiation by negatively regulating *PPARG* during adipogenesis. Collectively, these data suggest that offspring of obese mothers are at increased risk of obesity and metabolic disease	[73,74,75]
Diabetes	Different genes are involved in type 1 or type 2 diabetes mellitus, such as human leukocyte antigen (*HLA*)-*DQA1*, *HLA-DQB1*, *POMC*, insulin-like growth factor 2 (*IGF2*), insulin receptor (*INSR*), fat mass-and obesity-associated protein (*FTO*) as well as tumor necrosis factor (*TNF*), are either hypermethylated (*HLA-DQA1*, *HLA-DQB1*, *POMC*, *IGF2*, and *INSR*) or hypomethylated (*FTO* and *TNF*) in the promoter region of whole blood sample from offspring born after maternal bariatric surgery compared to before bariatric surgery	[76,77]
	Genes involved in immunological processes (including *TNF*-α, interleukin, major histocompatibility complex (MHC) class I and II signaling pathways) were differentially methylated in offspring of obese mothers.	[78,79]
	Several genes involved in immune response in patients with type 1 diabetes were hypermethylated in umbilical cord blood-derived monocytes, including signal transducer and activator of transcription 1 (*STAT1*), T cell receptors (*CD247*, *CD28*, and *CD3E*), MHC I class or II subunits (*HLADMB* and *HLA*-*DQB1*)	[79]
	Leptin promoters were hypermethylated in the placentas of obese mothers leading to decreased placental leptin expression while leptin deficiency is associated with hyperglycemia both in humans and animals	[80,81,82]
Chronic kidney disease (CKD)	Incremental increase in oxidative stress and mitochondrial impairment during the period of oocyte development laid down through epigenetic changes can also contribute to the transgenerational development of maternal obesity related CKD	[47,83]
	Maternal obesity correlates with global hypomethylation of key immune genes (T cells, cytokine, and chemokines) in umbilical cord blood-derived monocytes, such of which are involved in the development of inflammatory responses in kidney tissues and CKD pathology	[79]

## Data Availability

No new data were created or analyzed in this study. Data sharing is not applicable to this article.
